# Landslide hazard assessment: recent trends and techniques

**DOI:** 10.1186/2193-1801-2-523

**Published:** 2013-10-17

**Authors:** Sudhakar D Pardeshi, Sumant E Autade, Suchitra S Pardeshi

**Affiliations:** Department of Geography, University of Pune, Ganeshkhind, Pune 411007 India; Department of Geography, Swami Vivekanand Night College, Chattrapati Bhavan, Ayre Road, Dattanagar, Dombivli (E) 421201 India; Department of Geography, Annasaheb Magar College, Hadapsar, Pune 411028 India

**Keywords:** Landslide hazard, Landslide hazard zonation, Preparatory variables, Triggering mechanism, Geographical information system, Remotely sensed data

## Abstract

Landslide hazard assessment is an important step towards landslide hazard and risk management. There are several methods of Landslide Hazard Zonation (LHZ) viz. heuristic, semi quantitative, quantitative, probabilistic and multi-criteria decision making process. However, no one method is accepted universally for effective assessment of landslide hazards. In recent years, several attempts have been made to apply different methods of LHZ and to compare results in order to find the best suited model. This paper presents the review of researches on landslide hazard mapping published in recent years. The advanced multivariate techniques are proved to be effective in spatial prediction of landslides with high degree of accuracy. Physical process based models also perform well in LHZ mapping even in the areas with poor database. Multi-criteria decision making approach also play significant role in determining relative importance of landslide causative factors in slope instability process. Remote Sensing and Geographical Information System (GIS) are powerful tools to assess landslide hazards and are being used extensively in landslide researches since last decade. Aerial photographs and high resolution satellite data are useful in detection, mapping and monitoring landslide processes. GIS based LHZ models helps not only to map and monitor landslides but also to predict future slope failures. The advancements in Geo-spatial technologies have opened the doors for detailed and accurate assessment of landslide hazards.

## Introduction

Landslide is an important geological hazard that causes damage to natural and social environment. The concept of landslide is dealt by many authors differently. Varnes and IAEG (1984) defined landslides as ‘almost all varieties of mass movements on slope including some such as rock falls, topples and debris flow that involve little or no true sliding’. Brusden (1984) considered landslides as a unique form of mass transport and a process which do not require a transportation medium such as water, air or ice. Crozier (1986) defined landslides as ‘the outward and downward gravitational movement of the earth material without the aid of running water as a transporting agent’. According to Hutchinson (1988), ‘A landslide in its strict sense is a relatively rapid mass wasting process that causes the down slope movement of mass of rock, debris or earth triggered by variety of external stimulus’. A recent definition by Courture R (2011) simply states that ‘landslide is a movement of mass of soil (earth or debris) or rock down a slope’. This concept of landslide is more broaden with respect to the type of material that moves down slope.

Landslide causes loss of around 1000 lives and property worth $4 billion annually (EM-DAT 2007). According to the database created by the Centre for Research on Epidemiology of Disasters, landslides and related processes have killed over 61,000 people world over in the period between A.D. 1900 and A.D. 2009 (EMDAT 2010).

According to Brabb (1993), at least 90% of landslide losses can be avoided if the problem is recognized before the landslide event. Hence, there is a dire need for landslide hazard assessment at various spatial scales. The available literature needs to be reviewed to identify development in landslides hazard zonation methodologies world over. The present article reviews recent advances in landslide hazard assessment. The main focus of this paper is to discuss recent developments in landslide hazard zonation mapping methods, consideration of preparatory and triggering variables for mapping and application of Remote Sensing and Geographical Information System in the same. More than 100 recent research articles from referred journals viz. Geomorphology, Landslides, Engineering Geology, Natural Hazards and Earth Syst. Sci. and International Journal of Remote Sensing have been reviewed and compared on the basis of type of hazard zonation method adopted and variables considered for hazard zonation.

## Review

### Landslides and zonation

Landslides are natural events, but may turn into hazard and cause loss of lives and damage to man-made and natural structures. The term landslide hazard is defined by many authors differently, among them definition given by Burton et al. (1978), Rezig et al. (1996), Varnes and IAEG (1984), Cardinali et al. (2002), Guzzetti (2003) and Abella (2008) are important.

Though there are numerous approaches to define landslide hazards, many of the researchers have largely adopted or modified the definition given by Varnes and IAEG (1984).

### Methods of landslide hazard zonation

Landslide hazard zonation is an important step in landslide investigation and landslide risk management. Varnes and IAEG (1984) defines the term ‘zonation’ as ‘the process of division of land surface into areas and ranking of these areas according to the degree of actual or potential hazard from landslides or other mass movements’. Courture R (2011) explained the concept of landslide hazard as ‘division of land into somewhat homogeneous areas or domain and their ranking according to the degrees of actual or potential landslide susceptibility, hazard or risk or applicability of certain landslide related regulations’.

There has been significant growth both in landslide events particularly those induced by human activities and in number of landslide investigations in different parts of the world (Gutierrez et al. 2010). Gokceoglu and Sezer (2009) carried out statistical assessment of international landslide literature. They argued that publication of landslide related articles in the international journals has experienced exponential growth. They also pointed out that landslide susceptibility assessment is an important part of landslide investigation and has received more attention with highest number of publications in international journals.

Over last three decades LHZ mapping has been carried out in different parts of the world. Several approaches have been developed for LHZ mapping such as inventory based mapping, heuristic approach, probabilistic assessment, deterministic approach, statistical analysis and multi criteria decision making approach.

### Distribution (inventory) approach

Distribution analysis is one of the simplest qualitative approaches of LHZ mapping. It is also known as ‘landslide inventory’. In this analysis, landslide inventory maps are produced which portray spatial and temporal patterns of landslide distribution, type of movement, rate of movement, type of displaced material (earth, debris or rock) etc. Landslide data are obtained through field survey mapping, historical records, satellite images and aerial photo interpretation. Landslide distribution and density maps provide basis for other landslide susceptibility methods.

Cruden (1991) defined landslide inventory as ‘the simplest form of landslide information which records the location and where known, the date of occurrence, type of landslides that have left identifiable traces in the area’. Landslide inventory map also shows a slope failure by a single event or they may show cumulative effects of many events (Guzzetti et al. 2005a).

Landslide inventory play significant role in landslide hazard assessment. The quality and completeness of landslide inventory influences reliability of landslide investigation. Galli et al. (2008) compared landslide inventory maps prepared for different parts of Italy. Landslide distribution inventory, geomorphological landslide maps and multi-temporal landslide inventories were compiled and relationships among them were established. The results of the study revealed that complete landslide inventory map provide high predictive power for landslide susceptibility analysis.

Guzzetti et al. (2003) discussed three landslide event inventories and compared them using universal frequency-area statistics. They discussed the significance of completeness and resolution of landslide inventory maps in the landslide investigations. The results of the study portray that number of landslide events rapidly increased with increasing landslide area up to a maximum value and decreased as power law function.

Colombo et al. (2005) prepared landslide inventory by systematic surveys using aerial photo interpretation and GIS database to process the data using ARPA (Agenzia Regionale per la Protezione Ambientale - Regional Agency for Environmental Protection) archives. They classified landslides on the basis of landslide classification scheme by Varnes and IAEG (1984).

### Statistical approach

In last few years the approach towards LHZ has been changed from heuristic (knowledge based) approach to data driven approach (statistical approach) to minimize subjectivity in weightage assignment procedure and produce more objective and reproducible results Kanungo et al. (2009). Methods based on statistical analysis of geo-environmental factors related to landslide occurrence are preferred. The statistical methods for LHZ can be grouped into two viz. bi-variate statistical analysis and multi-variate statistical analysis.

### Bi-variate statistical analysis

The bi-variate statistical analysis for landslide hazard zonation compares each data layer of causative factor to the existing landslide distribution (Kanungo et al. 2009). Weights to the landslide causative factors are assigned based on landslide density. Frequency Analysis approach, Information Value Model (IVM), Weights of Evidence Model, Weighted overlay model etc. are important bi-variate statistical methods used in LHZ mapping.

### Weights of evidence model

A Weight of Evidence is a log linear form of Bayesian probability model for landslide susceptibility assessment that uses landslide occurrence as training points to derive prediction outputs. It calculates both unconditional and conditional probability of landslide hazards. This method is based on calculation of positive and negative weights to define degree of spatial association between landslide occurrence and each explanatory variables class. The Weights of Evidence model has been used for landslide susceptibility since 1990′s (Blahut et al. 2010). It uses different combinations of landslide causative factors in order to describe their interrelation with landslide distribution.

Blahut et al. (2010) applied WofE model to landslide susceptibility zonation mapping in Valtellina valley of central Italian Alps. The model was applied for different combinations of factor map. Four landslide susceptibility maps were prepared and compared using success rate curves. The best performing model was then selected with AUC (Area Under Curvature) value of 88%. Sterlacchini et al. (2011) carried out LHZ mapping in the Alpine environment of Italian Alps using WofE model. The model was validated by using success rate curves and prediction curves which gave success rate up to 88%. Piacentini et al. (2012) considered anthropogenic factors (land use and road network) for modeling landslide susceptibility using WofE method. Martha et al. (2013) applied this method to assess spatial landslide probability in Rudraprayag district of Garhwal Himalaya, India using semi-automatically created landslide inventories.

WofE a statistical method for landslide susceptibility modeling has proved to be a useful spatial data prediction model in many research works published in recent past (Piacentini et al. 2012; Schicker and Moon 2012; Martha et al. 2013; Neuhausev et al. 2012; Ghosh et al. 2009).

### Weighted overlay method

Weighted overlay is a simple bi-variate statistical method wherein weights are assigned based on the relationship of landslide causative factors with the landslide frequency. Sarkar et al. (1995) developed a methodology of LHZ for Rudrapeayag district in Garhwal Himalayas, India. Numerical weightages are assigned to causative factors on the basis of their relationships to the landslide frequency. Finally, the data layers were overlaid to produce LHZ map.

Panikkar and Subramaniyan (1997) carried out landslide hazard assessment using GIS based weighted overlay method in the area around Dehradun and Massori of Uttar Pradesh, currently Uttarakhand in India. The study revealed that rapid deforestation and urbanization have triggered landslides in the study area. This method is used to determine the relative importance of landslide causative factor in landslide occurrence (Parise 2002; Preuth et al. 2010; Cardinali et al. 2002).

Bi-variate discriminant function for ranking and weighting of landslide explanatory variables can be used effectively to produce landslide susceptibility map (Nagarajan et al. 2000).

### Frequency ratio approach

Frequency ratio is one of the bi-variate statistical approaches of landslide susceptibility assessment which is based on observed relationships between landslide distribution and each causative factor related to landslides. This method can be used to establish spatial correlation between landslide location and landslide explanatory factors (Lee 2005). Frequency ratio for each sub-class of individual causative factor is calculated based on their relationship with landslide occurrence. Landslide Susceptibility Index (LSI) is computed by summing of frequency ratio values of each factor.

Lee (2005) applied this model to landslide susceptibility in Penang region of Malaysia. He compared landslide susceptibility maps produced by Frequency Ratio Model and Logistic Regression model. Goswami et al. (2011) used frequency area statistics to assess spatial distribution of landslides in south west Calabria, Italy. Lee and Pradhan (2006) applied frequency ratio model to map landslide susceptibility for Penang region, Malaysia. The verification results showed 80.03% accuracy and found that incorporation of precipitation data in LHZ mapping improves prediction accuracy of landslide susceptibility map. Balteanu et al. (2010) applied this method to map Landslide Susceptibility in Romania.

### Information Value Method (IVM)

Information Value Model (IVM) is a bi-variate statistical method for spatial prediction of landslides based on relationships between landslide occurrence and related parameters (Sarkar et al. 2006). The information values are determined for each subclass of landslide related parameter on the basis of presence of landslide in a given mapping unit. Several studies have applied this method for LHZ mapping.

Zezere (2002) carried out landslide susceptibility assessment considering landslide typology in North Lisbon, Portugal. He found that information values for roads and fluvial channels are found in high landslide susceptibility class. The study revealed that anthropogenic activities play significant role in landslide occurrence and magnitude of landslides depend largely upon typology of landslides. Wang and Sassa (2005) compared landslide susceptibility maps for Minamata area of Japan produced by Logistic Regression and Information Value Model in GIS environment. Sarkar et al. (2006) presented a GIS based spatial data analysis for landslide hazard mapping in Sikkim Himalayas. They performed Information Value Model to integrate thematic data layers and subsequently numerical weights were assigned. Sharma et al. (2009) carried out GIS based landslide susceptibility zonation for Sikkim Himalayas using IVM. The accuracy assessment of landslide susceptibility map confirmed the model with highest degree of accuracy for high susceptibility class. Akbar and Ha (2011) developed an integrated model for landslide susceptibility zonation using Global Positioning System (GPS), Geographical Information System and Remotely Sensed data.

A modified form of pixel based information value model was applied to map landslide susceptibility. The study revealed that factors such as land use, rainfall intensity, distance from road and river influenced landslides more than that of other factors. Pereira et al. (2012) used IVM to evaluate the role of different combinations of landslide predisposing factors in the occurrence of shallow landslides in parts of Northern Portugal. IVM based 120 landslide susceptibility maps were produced and compared to determine ‘best fit model’ to landslide susceptibility in the study area. Recently, Balsubramani and Kumaraswamy (2013) applied this method for landslide hazard zonation mapping in Giri valley of Himachal Pradesh using high resolution satellite data.

Information Value Model has proved useful method in determining the degree of influence of individual causative factor responsible for landslide occurrence (Kanungo et al. 2009; Champatiray 2000; Champatiray et al. 2007; Arora et al. 2004).

### BIS based LHEF method

Bureau of Indian Standards (1998) has given guidelines for macro level landslide hazard zonation (BIS - IS 14496, Part 2) in India. BIS based Landslide Hazard Evaluation Factor (LHEF) rating scheme for landslide susceptibility zonation is a heuristic approach to landslide hazard assessment. According to Bureau of Indian Standards (1998) landslide hazard zonation procedure can be performed using LHEF rating for different landslide causative factors. BIS identified six landslide causative factors for hazard zonation viz. lithology, structure, slope morphometry, relative relief, land use-land cover and hydrological condition. In this method, the area under investigation is divided into small mapping units to which numerical weights are assigned for each thematic data layer and finally TEHD (Total Estimated Hazard) is obtained by adding weights of all variables for each mapping unit and Landslide Hazard map is produced.

Anbalagan et al. (2008) applied this method to map landslide susceptibility at meso-scale in Nainital, Kumaun Himalayas. The slope facet map was considered as base map to prepare thematic data layers. Few attempts have been made to apply this method in several parts of India (Naithani 2007; Singh et al. 2011; Champatiray et al. 2007 and Kannan et al. 2011).

BIS based LHEF rating scheme is a very simple and cost effective method of landslide hazard mapping. However, subjectivity in weight assignment procedure exists in this method which can affect the level of accuracy of hazard zonation map. Moreover, this method does not consider landslide distribution and therefore very difficult to test its validity.

Ghosh et al. (2009) evaluated effectiveness of the existing BIS method in Darjeeling Himalayas by adopting WofE model. They proposed a modified BIS model based on relationships of landslide causative factors with landslide distribution and found it more effective method for LHZ method.

### Fuzzy logic method

Fuzzy Logic method of landslide hazard zonation is based on bi-variate analysis wherein each landslide explanatory variable is represented by a value between 0 and 1 based on the degree of association of these parameters with landslide occurrence (Champatiray 2000). These membership values are then integrated using Fuzzy gama operator or Fuzzy Algebric Sum to produce landslide hazard zonation map. Champatiray et al. (2007) applied this method to landslide susceptibility assessment in Garhwal Himalayas.

Bi-variate statistical approach for LHZ mapping considers the relationship of landslide explanatory variables with landslide distribution. However, assigning weightage to the causative factors on the basis of this relationship may not always be appropriate as interrelationships among the causative factors also determine the degree of landslide hazard. Moreover, landslide events are outcome of several explanatory variables at a time. Therefore, it calls for application of multivariate statistical methods for more accurate LHZ mapping.

### Multi-variate statistical analysis

Multi-variate statistical analysis for landslide hazard zonation considers relative contribution of each thematic data layer to the total landslide susceptibility (Kanungo et al. 2009). These methods calculate percentage of landslide area for each pixel and landslide absence - presence data layer is produced followed by the application of multivariate statistical method for reclassification of hazard for the given area. Logistic regression model, Discriminant analysis, Multiple regression models, conditional analysis, Artificial Neural Networks (ANN) are commonly used methods for LHZ mapping.

### Logistic Regression (LR) analysis

The Logistic Regression is useful for predicting the presence or absence of a characteristic or outcome based on values of a set of predictor variables. This model is suited when dependent variable (e.g. landslide event) is dichotomous (Wang and Sassa 2005). Logistic Regression can be of two type viz. Binary Logistic (when dependent variable is dichotomous and independent variable is of any type) and Multinomial Logistic Regression (dependent variable with more than two classes). In case of landslide susceptibility mapping, the LR model find the best fitting model to describe the relationship between presence and absence of landslides and the set of independent variables such as slope angle, slope aspect, lithology and land use (Ayalew and Yamagishi 2005). It generates the model statistics and coefficient of formulae useful in defining susceptibility. If coefficient is positive, the landslide event is likely to occur. LR is a statistical model of slope instability built on the assumption that factor which caused slope failure in a region are the same as those which will generate landslides in future (Guzzetti et al. 1999).

Guzzetti et al. (1999) applied this method to model landslide susceptibility for Umbria region in central Italy. Rowbotham and Dudycha (1998) applied LR model to landslide susceptibility zonation for Hong Kong. They classified the region in terrain units based on Digital Elevation Model in GIS environment. Tolga et al. (2005) carried out landslide susceptibility assessment in Black Sea region of Turkey using LR model. They used Unique Condition Unit as a mapping unit for susceptibility classification.

Recently, several GIS based landslide susceptibility analyses using pixel as mapping unit have been applied. Several studies have applied LR model for LHZ mapping with comparatively high success rates (Chau et al. 2004; Wang and Sassa 2005; Ayalew et al. 2005; Garcia-Rodriguez et al. 2008; Ghosh 2011; Ohlmacher and Davis 2009; Hakan et al. 2008; Chang et al. 2007; Das et al. 2011; Akgun 2011; Mancini et al. 2010; Erner et al. 2010; Ayalew and Yamagishi 2005; Guzzetti et al. 1999; Das 2011; Atkinson and Massari 1998; Greco et al. 2007; Chang and Chiang 2009; Lee 2005; Atkinson and Massari 2011; Schicker and Moon 2012; Das et al. 2012; Meusburger and Alewell 2009; Lee et al. 2010; Dai and Lee 2002; Atkinson and Massari 2011Das et al. 2012; Xu et al. 2012).

### Discriminant analysis method

Discriminant analysis is one of the frequently used statistical models for LHZ. Discriminant analysis allows us to determine the maximum difference for each independent variable (e.g. landslide causative factor) between landslide group and non-landslide group and to determine weights for these factors (Lee et al. 2008). Slope units are classified into landslide affected and landslide free classes and then relative importance of each variable is expressed by computing Standardized Discriminant Function Coefficient (SDFC). SDFC show relative importance of each variable in discriminant function as a predictor of slope instability. Variable with high coefficient are strongly associated with presence or absence of landslide.

Several investigations for landslide susceptibility using Discriminant Analysis have been carried out in different parts of the world. Guzzetti et al. (2005b) applied Discriminant Analysis for landslide susceptibility zonation using 46 thematic variables in GIS environment. Percentage of landslide area and individual independent variable were computed for each pixel. Calvello et al. (2013) carried out landslide susceptibility zonation for Tammaro catchment of Southern Italy using Discriminant Analysis. They divided the region into hydrological units based on drainage network and geology of the area to define mapping unit. Terrain unit based classification for LHZ was done using Discriminant function.

Lee et al. (2008) used Discriminant Analysis (DA) for landslide hazard zonation mapping of central western Taiwan. The results indicated that slope gradient factor has highest coefficient and large percentage of weighting followed by NDVI. The success rate for landslide susceptibility map produced by this method was high (AUC = 0.9343). Ohlmacher and Davis (2009) prepared LHZ map using LR and DA in GIS environment for Kanas basin, USA. Eckhaut et al. (2009) applied DA landslide susceptibility assessment based on different mapping units. They compared landslide susceptibility maps produced by using multivariate approach for mapping unit viz. grid cells (pixel), Topographical Mapping Unit (TMU) and slope units. The study revealed that TMU based landslide susceptibility map show larger susceptible area than grid based LHZ map.

### Artificial neural network method

Landslides are governed by several preparatory and triggering factors which are complexly interrelated. The interrelationships between these factors and landslides are nonlinear in nature (Ercanglu 2005). To get accurate landslide susceptibility assessment more accurate methods are needed. ANN is a system based on the capability to learn a particular phenomenon similar to human being. ANN has over three layers of neurons which are connected by weights. This model use ‘Back propagation learning algorithm’ which define rules for assignment of weights. Weight of each variable is then adjusted to minimize errors. Artificial Neural Network (ANN) is a non-linear model and proved to be more effective in landslide hazard assessment (Catani et al. 2005; Ercanglu 2005; Pradhan and Lee 2009; Pradhan and Lee 2010; Bui et al. 2012).

Ercanglu (2005) produced landslide susceptibility map using ‘Back Propagation ANN model’ in NeuralNet module of Idrisi Kilimanjaro for west Black Sea, Turkey. He considered six parameters (slope gradient, aspect, topographical elevation, topographical shape, Wetness Index (WI) and Normalized Differential Vegetation Index (NDVI) for determination of weights in training phase of ANN model. The outcome of the model after validation indicated 82.5% correct results. Catani et al. (2005) applied ANN model to landslide susceptibility zonation in Arno river basin of central Italy. Landslide preparatory factor layers were overlaid to define Unique Condition Units (UCU). The final susceptibility map showed over 85% correctly recognized areas susceptible to landslides.

Chang and Liu (2004) performed ANN model for landslide susceptibility zonation in central Taiwan using high resolution satellite data. They argued that ANN method is better than Maximum Likelihood statistical method. Pradhan and Lee (2009) found 72-81% accurate results for landslide susceptibility in five training sites of Penang island, Malaysia. They applied ANN model in GIS environment. Pradhan and Lee (2010) applied this method for landslide susceptibility assessment in Malaysia. Bui et al. (2012) performed LHZ mapping in Malaysia using Bayesian Regularization Neural Networks and Levenberg Marquardt Neural Networks and found accuracy up to 90.3% and 86.1% respectively. Hence, ANN model can effectively be implemented in landslide hazard assessment in GIS environment to improve landslide prediction capability.

Arora et al. (2004) proposed ANN black box approach for landslide hazard zonation mapping. This approach determines weights which remain hidden during training stage. After training and testing of different neural network archives, the best one is selected based on the accuracy. They applied this model in Bhagirathi (Ganga) valley, India.

### Other multivariate techniques

Rotigliano et al. (2011) discussed the role of diagnostic areas in landslide susceptibility zonation mapping for Sicilian chain of Italy. The causative factors of landslides were combined to identify Unique Condition Units and then diagnostic areas were selected based on landslide types. Finally, the model was validated using prediction and success rate curves. selection of diagnostic areas is one of the most important steps in landslide hazard assessment to which unfortunately received very less attention (Rotigliano et al. 2011).

Clerici et al. (2002) applied conditional analysis method for landslide hazard zonation mapping in GIS environment using GRASS (Geographical Research Analysis Support System) commands. This method was applied to map landslide susceptibility for Parma river basin in Italian northern Apennines.

Multivariate approach to landslide susceptibility zonation hs widely been used since past few years and proved to be more objective method for assessing landslide hazards in complex geo-environmental settings (Conoscenti et al. 2008; Eckhaut et al. 2009; Ercanglu et al. 2003; Ayalew and Yamagishi 2005).

Application of Multi-variate statistical methods in LHZ mapping gives more accurate results but it includes complex calculations. These methods allow assessing comparative contribution of each causative factor in landslide occurrence. Therefore these methods are more objective in assignment of weightage in LHZ mapping procedure.

Few studies in recent times have assesed landslide susceptibility using systematic and extensive dendrogeomorphic mapping. Saez et al. (2012) have attempted to map probability of landslide reactivation using tree-ring records for landslide susceptibility assessment in south French Alps. They established relationship between landslide frequency and age structure of stand and disturbances using dendrogeomorphic analysis.

### Probabilistic approach

Probabilistic landslide hazard assessment helps to determine spatial, temporal and size probability of landslides (Guzzetti et al. 2005b). Probabilistic methods of LHZ mapping bring objectivity in assigning weights. In probabilistic approach to landslide susceptibility zonation, spatial distribution of landslides is compared with various explanatory variables within probabilistic framework (Kanungo et al. 2009). It includes Bayesian probability, certainty factor, favorability function etc. The degree of relationship between each thematic data layer with landslide distribution is transformed to a value based on probability distribution function. This approach is quantitative but certain degree of subjectivity exists in weight assignment procedure (Kanungo et al. 2009).

Guzzetti et al. (2005b) assessed landslide hazard in the Staffora River basin of north Apennines, Italy using probabilistic model. They computed probability of landslide size, temporal and spatial probability of landslides using frequency area distribution function. Poison probability model was applied to determine exceedance probability of landslide in each mapping unit.

Jaiswal et al. (2010a) carried out quantitative landslide hazard assessment along transport route in Nilgiri Hills, India. Frequency-volume statistics was performed to obtain probability of landslide magnitude for different return period. The results of the study indicated that variation in annual landslide frequency and volume are related to amount of rainfall. Therefore, probability of landslide size based on landslide frequency percentage can be estimated by incorporating rainfall magnitude data.

Das et al. (2011) assessed HSU (Homogeneous Susceptibility Unit) based landslide hazards using spatial, temporal and landslide size probabilities in Bhagirathi river basin of north Himalaya, India. A high resolution satellite datasets were used to define HSU for LHZ mapping. Recently, Jaiswal et al. (2010b) attempted to assess landslide susceptibility in Nilgiri hills, India using spatial probability to produce hazard and risk information for planning risk reduction measures. Further, they also developed a landslide early warning system based on rain fall database.

In recent times, several studies have attempted to apply probabilistic approach for quantitative landslide hazard zonation (Ghosh 2011; Floris and Bozzano 2008; Das 2011; Jaiswal and Van Westen 2013; Guzzetti et al. 2006; Polemio and Sdao 1999; Chelboard et al. 2006).

### Analytic hierarchy process approach

Landslide hazard assessment involves consideration of several landslide explanatory variables. It is a critical task to determine relative contribution of an individual parameter in landslide occurrence. Therefore, the application of Multi Criteria Decision making approach (MCDA) is of utmost importance in LHZ mapping. The Analytical Hierarchy Process (AHP) is a multi criteria decision making process of measurement through pair wise comparisons and relies on the judgements of the experts to derive priority scales (Saaty 2008). AHP operates at four levels viz. defining problem, determination of goals and alternatives, construction of pair wise comparison matrix, determining weights and obtaining overall priority. In LHZ, different landslide causative factors are considered as alternatives. Absolute numbers (from 1 to 9) are assigned to each landslide related parameter based on its relative importance and comparison matrices are constructed to compute Consistency Ratio (CR) and Consistency Index (CI). Akgun (2011) compared landslide hazard maps produced by Logistic Regression (LR), Multi Criteria Decision Approach (MCDA) and Likelihood Ratio Method (LRM) for Azmir, Turkey using AUC (Area Under Curvature) method. The correlation coefficients (r) were found to be 0.86, 0.62, 0.58 for LR*LRM, LR*MCDA and LRM*MCDA respectively. The LRM and MCDA showed similar results. Ayalew et al. (2005) compared LSZ maps using LR and AHP model to assess landslide hazard. The study revealed that if there is increase in number of susceptibility classes, LR model gives more details than AHP. However when these maps compared with landslide activity map, AHP based map performed better than LR model. In recent times, several attempts have been made to apply GIS based AHP to map landslide susceptibility in various parts of the world (Mondal and Maiti 2012; Ma et al. 2013; Kavzoglu et al. 2013).

### Rainfall threshold model

Rainfall threshold for landsliding refers to minimum intensity or duration of rainfall necessary to cause landslide (Varnes and IAEG 1984). Cumulative rainfall, antecedent rainfall, rainfall intensity and rainfall duration are most commonly used parameters to design rainfall threshold. The critical rainfall threshold model (Qcr) is based on soil properties, slope angle, upslope drainage, wet soil bulk density and density of water. Several studies on landslide susceptibility assessment have used rainfall threshold model to predict landslide. The rainfall threshold decreases with increasing seasonal accumulation and become constant at 11 mm/day Gabet et al. (2004).

Chelboard et al. (2006) applied cumulative rainfall threshold (CT) for prediction of landslides in Seattle, Washington, USA. The model was compared with historical records of rainfall and landslide events. The results indicated that CT captured over 90% of the historical landslide events. They argued that both CT and exceedance rainfall intensity duration threshold must be used together for landslide prediction.

Floris and Bozzano (2008) proposed a modification in conventional rainfall threshold model for landslide hazard assessment. Based on historical records of landslide events and rainfall, rainfall exceedence thresholds were estimated for two complex landslides in south Apennines, Italy. Chang and Chiang (2009) proposed an integrated model for landslide susceptibility combining deterministic, statistical and rainfall threshold model for typhoon induced landslides in Taiwan. Gabet et al. (2004) applied rainfall threshold for landslides in Nepal Himalaya considering daily and seasonal rainfall threshold for modeling. They suggested that sufficient antecedent rainfall is necessary to produce positive pore pressure and trigger landslides.

Dahal and Hasegawa (2008) studied over 670 landslides occurred from 1951 to 2006 in Nepal Himalaya to analyze rainfall threshold. Coe et al. (2004), Polemio and Sdao (1999) have also applied rainfall threshold for landslide susceptibility assessment.

### Physically-based landslide susceptibility models

Physically based models for landslide hazard assessment describes physical processes leading to the landslide event and are based on simple mechanical laws. These models account for the transient ground water response of slope to rainfall (Kuriakose 2010). These models do not need long term landslide data and therefore can also be applicable to the areas with incomplete landslide inventories (Kuriakose 2010).

Salciarini et al. (2006) applied the Transient Rainfall Infiltration and Grid based Slope Stability (TRIGRS) model for modelling rainfall induced shallow landslides in central Umbria region of central Italy. They have chosen known rainfall events and past landslide records to calibrate the model and simulations were performed. They argued that high resolution digital elevation models and information about spatial distribution of physical properties of the surface are needed for better simulation in TRIGRS model.

The real time susceptibility to shallow landslides has been assessed by Montrasio et al. (2011) for Emilion Apennine in north Italy. They compared SLIP (shallow Landslide Instability Prediction) and TRIGRS (Transient Rainfall Infiltration and Grid based Slope Stability) models of landslide susceptibility analysis in GIS (Geographical Information System) environment. The results of the study indicates that both the models have similar predictive capability.

Kuriakose (2010) carried out a detailed study to compare four physically based models viz. SHALSTAB (Shallow Landsliding Stability), SINMAP (Stability INdex MAPping), TRIGRIS and STARWAR+PROBSTAB (Storage and Redistribution of Water on Agricultural and Revegetated Slope + PROBability of STABility) models in Western Ghats of Kerala, India. The study revealed that STARWAR+PROBSTAB model is the most suitable model for assessment of spatio-temporal probabilities of shallow landslides.

Recently, the High Resolution Slope Stability Simulator (HIRESSS) model was used to predict landslides based on hydrological parameters (Mercogliano et al. 2013). The study also incorporated Global Circulation Model for analysis of rainfall parameters.

### Application of RS and GIS in LHZ

Extraction of relevant spatial information related to landslide occurrence is an integral part of hazard assessment. Remotely Sensed (RS) data combined with Geographical Information System (GIS) are proved to be effective tools for generating and processing spatial information. The advancement in earth Observation (EO) techniques facilitate effective landslide detection, mapping, monitoring and hazard analysis (Tofani et al. 2013).

The review of few studies on landslide hazard assessment using RS data indicate that aerial photographs are widely used in landslide detection and mapping (Galli et al. 2008; Guzzetti et al. 2003; Yeon et al. 2010; Rotigliano et al. 2011; Guzzetti et al. 2005a; Rowbotham and Dudycha 1998; Pradhan and Lee 2009; Panikkar and Subramaniyan 1997; Miller and Burnett 2007; Ayalew and Yamagishi 2005; Chau et al. 2004; Clerici et al. 2002). Good quality aerial photographs help in accurate landslide detection and mapping. However, aerial photographs may not be used in continuous landslide monitoring, since it does not prove repetitive coverage of the same area.

The recent developments in the application of satellite RS data in landslide studies in Europe has been discussed by Tofani et al. (2013). The study showed that over 70% of the total applications of RS data for landslide studies are owned by landslide detection, mapping and monitoring. High resolution satellite data are being effectively used for landslide detection, mapping, monitoring and other applications (Gomez et al. 2000; Saraf et al. 2009; Akbar and Ha 2011; Naithani 2007; Nagarajan et al. 2000; Ma et al. 2013; Mondal and Maiti 2012; Balsubramani and Kumaraswamy 2013 and Chand 2008).

Use of Digital Elevation Model (DEM) is of immense importance in landslide hazard assessment. Several thematic data layers such as slope angle, slope aspect, curvature, lineaments, drainage, ridges etc. can be extracted from DEM with good resolution. Landslide hazard zonation studies in recent times have used DEM with high resolution to generate spatial information data layers related to landslide hazards (Gomez et al. Saraf et al. 2009; Dahl et al. 2010; Yeon et al. 2010; Akbar and Ha 2011; Naithani 2007; Jaiswal et al. 2010b; Rotigliano et al. 2011; Nagarajan et al. 2000; Guzzetti et al. 2005b; Rowbotham and Dudycha 1998; Barla et al. 2010; Leroi 1996; Miller and Burnett 2007; Ma et al. 2013; Ayalew and Yamagishi 2005; Balsubramani and Kumaraswamy 2013; Ghosh et al. 2009; Calvello et al. 2013; Tolga et al. 2005; Clerici et al. 2002; Coe et al. 2004; Jelinek and Wagner 2007; Chand 2008; Ruff and Czurda 2008). Few studies also used radar techniques (e.g. DInSAR, PSInSAR) for landslide hazard assessment (Barla et al. 2010; Catani et al. 2005).

Geographical Information System is widely used in landslide hazard assessment especially for generation of thematic data layers, computation of different indices, assignment of weights, data integration and generation of LSZ maps. Several LSZ methods such as ANN, Decision Tree model, Weighted Overlay, AHP, MCDA, IVM and physically based landslide hazard models are GIS based models to predict landslide probability (Chang and Liu 2004; Saraf et al. 2009; Yeon et al. 2010; Pradhan and Lee 2009; Kavzoglu et al. 2013; Akgun 2011; Ayalew et al. 2005; Mondal and Maiti 2012; Ma et al. 2013).

## Conclusions

Landslide hazard zonation is a critical task in landslide management process. Landslides are influenced by several preparatory and triggering factors which vary significantly from region to region. It is therefore difficult to determine weights for given parameter. The assignment weights based on relative importance of landslide causative factors is determined by several LHZ methods differently. Heuristic and semi quantitative techniques involve subjectivity in assigning of weights therefore validity of these maps cannot be assessed. Quantitative methods on the other hand, provide objective methods for determining weights for a given parameter based on their relationships with landslide occurrence. Multi-criteria decision approach provides tools to determine weights based on pair wise comparison. Application of Remote Sensing and Geographical Information System is of immense importance for effective landslide hazard assessment. High resolution satellite data combined with powerful GIS techniques have improved the level of accuracy of LHZ maps in recent times.

## References

[CR1] Abella E (2008). Multi-scale landslide risk assessment in Cuba. international institute for geo-information science and earth observation.

[CR2] Akbar T, Ha S (2011). Landslide hazard zoning along Himalaya Kaghan Valley of Pakistan-by integration of GPS, GIS, and remote sensing technology. Landslides.

[CR3] Akgun A (2011). Acomparison of landslide susceptibility maps produced by logistic regression, multi-criteria decision, and likelihood ratio methods: a case study at Lzmir, Turkey. Landslide.

[CR4] Anbalagan R, Chakraborty D, Kohali A (2008). Landslide hazard zonation (LHZ) mapinh on meso-scale for systematic town planning in mountainous terrain. J Sci Ind Res.

[CR5] Arora M, Das Gupta A, Gupta R (2004). An artificial neural network approach for landslide hazard zonation in the Bhagirathi (Ganga) Valley, Himalaya. Int J Remote Sensing.

[CR6] Atkinson P, Massari R (1998). Generalised linear modelling of susceptibility to landsliding in the Central Apennines Italy. Compt Rendus Geosci.

[CR7] Atkinson P, Massari R (2011). Autologistic modelling of susceptibility to landsliding in the Central Apennines, Italy. Geophys J Roy Astron Soc.

[CR8] Ayalew L, Yamagishi H (2005). The application of GIS-based logistic regression for landslide susceptibility mapping in the Kakuda-Yahiko Mountains, Central Japan. Geophys J Roy Astron Soc.

[CR9] Ayalew L, Yamagishi H, Marui H, Kanno T (2005). Landslides in Sado Island of Japan: Part II GIS-based susceptibility mapping with comparisons of results from two methods and verifications. Eng Geol.

[CR10] Balsubramani K, Kumaraswamy K (2013). Application of geospatial technology and information value technique in landslide hazard zonation mapping: a case study of Giri Valley, Himachal Pradesh. Disaster Advances.

[CR11] Balteanu D, Chendes V, Sima M, Enciu P (2010). A country-wide spatial assessment of landslide susceptibility in Romania. Geophys J Roy Astron Soc.

[CR12] Barla G, Antolini F, Barla M, Mensi E, Piovano G (2010). Monitoring of the Beauregard landslide (Aosta Valley, Italy) using advanced and conventional techniques. Eng Geol.

[CR13] Blahut J, VanWesten C, Sterlacchini S (2010). Analysis of landslide inventories for accurate prediction of debris-flow source areas. Geophys J Roy Astron Soc.

[CR14] Brabb E (1993). Proceedings of 7th International Conference and field workshop on landslide in Czech and Slovak Republics. Proposal for worldwide landslide hazard maps.

[CR15] Brusden D, Brusden D, Prior D (1984). Mudslides. Slope Instability.

[CR16] Bui D, Pradhan B, Lofman O, Dick O (2012). Landslide susceptibility assessment in the Hoa Binh Province of Vietnam: a comparison of the Levenberg-Marquardt and Bayesian regularized neural networks. Geophys J Roy Astron Soc.

[CR17] Bureau of Indian Standards (1998). Preparation of landslide hazard zonation maps in mountainous terrain - Guidelines (Part2-Macrozonation), vol 14496.

[CR18] Burton I, Kates R, White G (1978). The environment as hazard.

[CR19] Calvello M, Cascini L, Mastroianni S (2013). Landslide zoning over large areas from a sample inventory by means of scale-dependant terrain units. Geophys J Roy Astron Soc.

[CR20] Cardinali M, Reichenbach P, Guzzetti F, Ardizzone F, Antonini G, Galli M, Cacciano M, Castellani M, Salvati P (2002). A geomorphological approach to estimation of landslide hazards and risks in Umbria, Central Italy. Nat Hazards Earth Syst Sci.

[CR21] Catani F, Casagil N, Ermini L, Righini G, Menduni G (2005). Landslide hazard and risk mapping at catchment scale in the Arno River basin. Landslides.

[CR22] Champatiray P, Roy P, Van Westen C, Jha V, Lakhera R (2000). Perationalization of cost-effective methodology for landslide hazard zonation using RS and GIS: IIRS initiative. Natural Disasters and their mitigation; Remote Sensing and Geographical Information System Perspectives.

[CR23] Champatiray P, Dimri S, Lakhera R, Sati S (2007). Fuzzy based methods for landslide hazard assessment in active seismic zone of Himalaya. Landslides.

[CR24] Chand D (2008). Landslide monitoring in space and time using optical satellite imagery and DEM derived parameters: case study from Garhwal Himalaya, Uttarakhand, India.

[CR25] Chang K, Chiang S (2009). An integrated model for predicting rainfall induced Landslides. Geophys J Roy Astron Soc.

[CR26] Chang K, Liu J (2004). Geo-Imagery Bridging continents. Landslide features interpreted by neural network method using a high resolution satellite image and digital topographical data.

[CR27] Chang K, Chiang S, Hsu M (2007). Modelling typhoon- and earthquake-induced landslide in a mountainous watershed using logistic regression. Geophys J Roy Astron Soc.

[CR28] Chau K, Sze Y, Fung M, Wong E, Fong E, Chan L (2004). Landslide hazard analysis for Hong Kong using landslide inventory and GIS. Compt Rendus Geosci.

[CR29] Chelboard F, Baum R, Godt J (2006). Rainfall Thresholds for Forecasting Landslides in the Seattle, Washington, Area- Exceedance and Probability.

[CR30] Clerici A, Perego S, Tillini C, Vescovi P (2002). A procedure foe landslide susceptibility zonation by the conditional analysis method. Geophys J Roy Astron Soc.

[CR31] Coe J, Godt J, Baum R, Buchnam R, Michael J, Lacerda W, Ehrlich M, Fontura S, Sayao A (2004). Landslide susceptibility from topography in Guatemala. Landslides: Evaluation and stabilization.

[CR32] Colombo A, Lanteri L, Ramasco M, Troisi C (2005). Systematic GIS based landslide inventory as the first step for effective landslide hazard management. Landslides.

[CR33] Conoscenti C, Di Maggio C, Rotigliano E (2008). GIS analysis to assess landslide susceptibility in a fluvial basin of NW Sicily (Italy). Geophys J Roy Astron Soc.

[CR34] Courture R (2011). Landslide Terminology - National Technical Guidelines and Best Practices on Landslides. Geological Survey of Canada.

[CR35] Crozier M (1986). Landslides-causes, consequences and environment.

[CR36] Cruden D (1991). A simple definition of a landslide. Bull-Int Assoc Eng Geology.

[CR37] Dahal R, Hasegawa S (2008). Representative rainfall thresholds for landslides in the Nepal Himalaya. Geophys J Roy Astron Soc.

[CR38] Dahl M, Mortensen L, Veihe A, Jensen N (2010). A simple qualitative approach for mapping regional landslide susceptibility in the Faroe Islands. Nat Hazards Earth Syst Sci.

[CR39] Dai F, Lee C (2002). Frequency-volume relation and prediction of rainfall-induced landslide. Eng Geol.

[CR40] Das I (2011). Spatial Statistical Modelling for assessing landslide hazard and vulnerability.

[CR41] Das I, Stein A, Kerle N, Dadhwal V (2011). Probabilistic landslide hazard assessment using homogeneous susceptible units (HSU) along a national highway corridor in the northern Himalayas, India. Landslides.

[CR42] Das I, Stein A, Kerle N, Dadhwal V (2012). Landslide susceptibility mapping along road corridors in the Indian Himalayas using Bayesian logistic regression models. Geophys J Roy Astron Soc.

[CR43] Eckhaut M, Reichenbach P, Guzzetti F, Rossi M, Poesen J (2009). Combined landslide inventory and susceptibility assessment based on different mapping units: an example from the Flemish Ardennes, Belgium. Nat Hazards Earth Syst Sci.

[CR44] EM-DAT (2007). Emergency Disasters Data Base, Volume 2007: Brussels, Belgium, Centre for Research on the Epidemiology of Disasters.

[CR45] EMDAT (2010). Emergency Disasters Data Base, Volume 2010: Brussels, Belgium, Centre for Research on the Epidemiology of Disasters.

[CR46] Ercanglu M (2005). Landslide susceptibility assessment of SE Bartin (West Black Sea region, Turkey) by artificial neural networks. Nat Hazard Earth Syst Sci.

[CR47] Ercanglu M, Gokceoglu C, Van Asch W (2003). Landslide Susceptibility Zoning of North of Yenice (NW Tuekey) by Multivariate Stastical Techniques.

[CR48] Erner A, Sebnem H, Duzgun B (2010). Improvement of statistical landslide susceptibility mapping by using spetial and global regression method in the case of More and Romsdal (Norway). Landslides.

[CR49] Floris M, Bozzano F (2008). Evaluation of landslide reactivation: a modified rainfall threshold model based on historical records of rainfall and landslides. Geophys J Roy Astron Soc.

[CR50] Gabet E, Burbank D, Putkonen J, Pratt-Situala B, Ojha T (2004). Rainfall thresholds for landsliding in the Himalaya of Nepal. Geophys J Roy Astron Soc.

[CR51] Galli M, Ardizzone F, Cardinali M, Guzzetti F, Reichenbach P (2008). Comparing landslide inventory maps. Geophys J Roy Astron Soc.

[CR52] Garcia-Rodriguez M, Malpica J, Benito B, Diaz M (2008). Susceptibility assessment of earthquake-triggered landslides in El Salvador using logistic regression. Geophys J Roy Astron Soc.

[CR53] Ghosh S (2011). Knowledge Guided Empirical Prediction of Landslide Hazard.

[CR54] Ghosh S, Van Westen C, Carranza E, Ghoshal T, Sarkar N, Surendranath M (2009). A quantitative approach for improving BIS(Indian) method of medium-scale landslide susceptibility. J Geological Soc India.

[CR55] Gokceoglu C, Sezer E (2009). A statistical assessment on international landslide literature (1945–2008). Landslides.

[CR56] Gomez H, Bradshow R, Mather P, Casanova E (2000). Monitoring the distribution of shallow landslide prone areas using Remote Sensing, DTM and GIS - a case study from the tropical Andes of Venezuela. Remote Sensing in 21st century: Economic and Environmental applications.

[CR57] Goswami R, Mitchell N, Brocklehurst S (2011). Distribution and causes of landslides in the eastern Peloritani of NE Sicily and western Aspromonte of SW Calabria, Italy. Geophys J Roy Astron Soc.

[CR58] Greco R, Sorriso-Valvo M, Catalano E (2007). Logistic regression analysis in the evaluation of mass movement susceptibility: the aspromonte case study, Calabria, Italy. Eng Geol.

[CR59] Gutierrez F, Soldati M, Audemard F, Balteanu D (2010). Recent advances in landslide investigation: issues and perspectives. Geophys J Roy Astron Soc.

[CR60] Guzzetti F (2003). Proceedings of the 4th EGS Plinius Conference held at Mallorca, Spain. Landslide Hazard Assessment and Risk Evaluation: Limits and Perspectives.

[CR61] Guzzetti F, Carrara A, Cardinali M, Reichenbach P (1999). Landslide hazard evaluation: a review of current techniques and their application in a multi-study, Central Italy. Geophys J Roy Astron Soc.

[CR62] Guzzetti F, Aleotti P, Malamud B, Turcotte DL (2003). Proceedings of 4th EGS Plinius Conference held at Mallorca. Comparison of three landslide event inventories in Central and Northern Italy.

[CR63] Guzzetti F, Reichenbach P, Cardinali M, Ardizzone F (2005). Probabilistic landslide hazard assessment at the basin scale. Geophys J Roy Astron Soc.

[CR64] Guzzetti F, Reichenbach P, Cardinali M, Galli M, Ardizzone F (2005). Probablistic landslide hazard assessment at the basin scale. Geophys J Roy Astron Soc.

[CR65] Guzzetti F, Galli M, Reichebach P, Ardizzone F, Cardinali M (2006). Landslide Hazard assessment in the Collazzone area, Umbria, Central Italy. Nat Hazards Earth Syst Sci.

[CR66] Hakan A, Tamer Y, Serap D (2008). Landslide susceptibility mapping for a part of tectonic Kelkit Valley (Eastern Black Sea Region of Turkey). Geophys J Roy Astron Soc.

[CR67] Hutchinson J, Fairbridge R (1988). Mass movement. The Encyclopedia of Geomorphology. Reinold.

[CR68] Jaiswal P, Van Westen C (2013). Use of quantitative landslide hazard and risk information for local disaster risk reduction along a transportation corridor: a case study from Nilgiri District, India. Nat Hazards.

[CR69] Jaiswal P, van Westen C, Jetten V (2010). Quantitative assessment of landslide hazard along transportation lines using historical records. Landslides.

[CR70] Jaiswal P, van Westen C, Jetten V (2010). Quantitative assessment of direct and indirect landslide risk along transportation lines in southern India. Nat Hazards Earth Syst Sci.

[CR71] Jelinek R, Wagner P (2007). Landslide hazard zonation by deterministic analysis. Landslides.

[CR72] Kannan M, Saranathan E, Anbalagan R (2011). Macro landslide hazard zonation mapping - case study from bodi-bodimettu Ghats section, Theni District, Tamil Nadu, India. J Indian Soc Remote Sensing.

[CR73] Kavzoglu T, Sahin E, Colkensen I (2013). Landslide susceptibility mapping using GIS based multi-criteria decision analysis, support vector machines and logistic regression. Landslides.

[CR74] Kanungo D, Arrora M, Sarkar S, Gupta R (2009). Landslide Susceptibility Zonation (LSZ) mapping-a review. J South Asia Disaster Stud.

[CR75] Kuriakose S (2010). Physically-based dynamic modeling of the effect of land use changes on shallow landslide initiation in the Western Ghats of Kerala.

[CR76] Lee S (2005). Application of logistic regression model and its validation for landslide susceptibility mapping using GIS and remote sensing data. Int J Remote Sens.

[CR77] Lee S, Pradhan B (2006). Probabilistic landslide hazard and risk mapping on Penang Island, Malaysia. J Earth Syst Sci.

[CR78] Lee C, Huang C, Lee J, Pan K, Lin M, Dong J (2008). Statistical approach to storm event-induced landsliZ des susceptibility. Nat Hazards Earth Syst Sci.

[CR79] Lee S, Yu T, Peng W, Wang C (2010). Incorporating the effect of topographic amplification in the analysis of earthquake-induced landslide hazards using logistic regression. Nat Hazards Earth Syst Sci.

[CR80] Leroi E, Senneset K (1996). Landslide hazard - risk maps at different scales: objectives, tools and developments. Landslides Glissements de terrain.

[CR81] Ma F, Wang J, Yuan R, Zhao H, Guo J (2013). Application of analytical hierarchy process and least square method for landslide susceptibility assessment along the Zhong - Wu natural gas pipelines. China. Landslides. Doi:.

[CR82] Mancini F, Ceppi C, Ritrovato G (2010). GIS and statistical analysis for landslide susceptibility mapping in the Daunia area, Italy. Nat Hazards Earth Syst Sci.

[CR83] Martha T, vanWesten C, Kerle N, Jetten V, Vinod Kumar K (2013). Landslide hazard and risk assessment using semi-automatically created landslide inventories. Geophys J Roy Astron Soc.

[CR84] Mercogliano P, Segoni S, Rossi G, Sikorsky B, Tofani V, Schiano P, Catani F, Casagli N (2013). Brief communication “A prototype forecasting chain for rainfall induced shallow landslides”. Nat Hazards Earth Syst Sci.

[CR85] Meusburger K, Alewell C (2009). On the influence of temporal change om the validity of landslide susceptibility maps. Nat Hazards Earth Syst Sci.

[CR86] Miller D, Burnett K (2007). Effects of forest cover, topography and sampling extent on the measured density of shallow translational landslides. Water Resour Res.

[CR87] Mondal S, Maiti R (2012). Landslide susceptibility analysis of Shiv-Khola Watershed, Darjiling; a remote sensing and GIS based Analytic Hierarchy Process. J Indian Soc Remote Sensing.

[CR88] Montrasio L, Valentino R, Losi G (2011). Towards a real-time susceptibility assessment of rainfall-induced shallow landslides on a regional scale. Nat Hazards Earth Syst Sci.

[CR89] Nagarajan R, Roy A, Vinodkumar R, Khire M (2000). Landslide hazard susceptibility mapping based on terrain and climatic factors for tropical monsoon region. Eng Geol.

[CR90] Naithani A (2007). Macro landslide hazard zonation mapping using uni-variate statistical analysis in parts of Garhwal Himalaya. J Geological Soc India.

[CR91] Neuhausev B, Damm B, Terhorst B (2012). GIS based assessment of landslide susceptibility on the basis of weights of evidence model. Landslides.

[CR92] Ohlmacher G, Davis J (2009). Using multiple logistic regression and GIS technology to predict landslide hazard in northeast Kansas, USA. Eng Geol.

[CR93] Panikkar S, Subramaniyan V (1997). Landslide hazard analysis of the area around Dehra Dun and Mussoorie, Uttar Pradesh. Curr Sci.

[CR94] Parise M (2002). Landslide hazard zonation of slopes susceptible to rock falls and topples. Nat Hazards Earth Syst Sci.

[CR95] Pereira S, Zezere J, Bateira C (2012). Assessing predictive capacity and conditional independence of landslide predisposing factors for shallow landslide susceptibility models. Nat Hazards Earth Syst Sci.

[CR96] Piacentini D, Troini F, Soldati M, Notamicola C, Saveli D, Scheneiderbauer S, Strada C (2012). Statistical analysis for assessing shallow-landslide susceptibility in South Tyrol (south-eastern Alps, Italy). Geophys J Roy Astron Soc.

[CR97] Polemio M, Sdao F (1999). The role of rainfall in the landslide hazard: the case study of the Avigliano Urban area (Southern Apenniens Italy). Eng Geol.

[CR98] Pradhan B, Lee S (2009). Landslide risk analysis using artificial neural network model focussing on different training sites. Int J Phys Sci.

[CR99] Pradhan B, Lee S (2010). Regional landslide susceptibility analysis using back-propagation neural network model at Cameron Highland, Malaysia. Landslides.

[CR100] Preuth T, Glade T, Demoulin A (2010). Stability analysis of a human-influenced landslides in eastern Belgium. Geophys J Roy Astron Soc.

[CR101] Rezig S, Favre J, Leroi E, Sennset (1996). The probabilistic evaluation of landslide risk. Landslides.

[CR102] Rotigliano E, Angesi V, Cappadonia C, Conoscenti C (2011). The role of diagnostic areas in the assessment of landslide susceptibility model: a test in the sicillan chain, Italy. Nat Hazards.

[CR103] Rowbotham D, Dudycha D (1998). GIS modelling of slope stability in Phewa Tal watershed, Nepal. Geophys J Roy Astron Soc.

[CR104] Ruff M, Czurda K (2008). Landslide susceptibility analysis with a heuristic approach in the Eastern Alps (Vorarlberg, Austria). Geophys J Roy Astron Soc.

[CR105] Saaty T (2008). Decision making with the analytical hierarchy process. Int J Services Sci.

[CR106] Salciarini D, Godt J, Savage W, Conversini P, Baum R, Michel J (2006). Modeling regional initiation of rainfall-induced shallow landslide in the eastern Umbria Region of central Italy. Landslides.

[CR107] Saraf A, Das J, Rawat V (2009). Satellite based detection of early occurring of co-seismic landslides. J South Asia Disaster Stud (Journal of SAARC Disaster Management Centre).

[CR108] Sarkar S, Kanungo D, Mehrotra G (1995). Landslide hazard zonation: a case study of garhwal Himalaya, India. Mt Res Dev.

[CR109] Sarkar S, Kanungo D, Patra A, Kumar P (2006). Disaster mitigation of debris flows, slope failures and landslides. GIS based landslide susceptibility mapping- a case study in Indian Himalaya.

[CR110] Schicker R, Moon V (2012). Comparison of bivariate and multivariate statistical approaches in landslide susceptibility mapping at a regional scale. Geophys J Roy Astron Soc.

[CR111] Saez J, Corona C, Stoffel M, Schoeneich P, Berger F (2012). Probability maps of landslide reactivation derived from tree-ring records: Pra Bellon landslide, southern French Alps. Geophys J Roy Astron Soc.

[CR112] Sharma L, Patel N, Ghosh M, Debnath P (2009). Geographical Information System Based Landslide Probabilistic Model with Trivariate approach– A Case Study in Sikkim Himalaya. Eighteenth United Nations Regional Cartographic Conference for Asia and the Pacific.

[CR113] Singh C, Behra K, Rocky W (2011). Landslide susceptibility along NH-39 between Karong and Mao, Senapati District, Manipur. J Geological Soc India.

[CR114] Sterlacchini S, Ballabio C, Blahut J, Masetti M, Sorichetta A (2011). Spatial agreement of predicted patterns in landslide susceptibility maps. Geophys J Roy Astron Soc.

[CR115] Tofani V, Segoni S, Agostini A, Catani F, Casagli N (2013). Use of remote sensing for landslide studies in Europe. Nat Hazards Earth Syst Sci.

[CR116] Tolga C, Hakan A, Candan G, Harun S, Tamer Y (2005). Susceptibility assessment of shallow earth flows triggered by heavy rainfall at three catchments by logistic regression analyses. Geophys J Roy Astron Soc.

[CR117] Varnes D, IAEG (1984). Landslide hazard zonation: a review of principles and practice.

[CR118] Wang H, Sassa K (2005). Comparative evaluation of landslide susceptibility in Minamata area, Japan. Environ Geol.

[CR119] Xu C, Dai F, Xu X, Lee Y (2012). GIS-based support vector machine modeling of earthquake-triggered landslide susceptibility in the Jianjiang River watershed, China. Geophys J Roy Astron Soc.

[CR120] Yeon Y, Han J, Ryu K (2010). Landslide susceptibility mapping in Injae, Korea using a decision tree. Eng Geol.

[CR121] Zezere J (2002). Landslide susceptibility assessment considering landslide typology. A case study in the area north of Lisbon (Portugal). Nat Hazard Earth Syst Sci.

